# Primary Ventral Hernia Repair and the Risk of Postoperative Small Bowel Obstruction: Intra Versus Extraperitoneal Mesh

**DOI:** 10.3390/jcm12165341

**Published:** 2023-08-16

**Authors:** Marine Goullieux, Fawaz Abo-Alhassan, Remi Vieira-Da-Silva, Papet Lauranne, Adeline Guiraud, Pablo Ortega-Deballon

**Affiliations:** Department of Digestive Surgery, University Hospital of Dijon, 14 Rue Paul Gaffarel, 21000 Dijon, France

**Keywords:** ventral hernia, intestinal occlusion, hernia, mesh

## Abstract

Objective: The aim of this study was to compare the likelihood of bowel obstruction according to the placement of the mesh (either intraperitoneal or extraperitoneal) in ventral hernia repairs. Materials and methods: Patients were divided into two groups, an intraperitoneal (IP) group (mesh placed by laparoscopy or with an open approach) and an extraperitoneal (EP) group, all operated on in the Digestive Surgery Department at the Dijon University Hospital. The primary outcome was the occurrence of an episode of bowel obstruction requiring hospitalization and confirmed by abdominal CT scan. Results: Between March 2008 and July 2021, 318 patients were included, with 99 patients in the EP group (71 meshes placed preperitoneally and 28 placed retromuscularly) and 219 patients in the IP group (175 patients operated on laparoscopically versus 44 patients by direct approach). Three patients presented an episode of acute intestinal obstruction, with no difference between the two groups (*p* = 0.245), although all bowel obstructions occurred in the IP group and with the laparoscopic approach (1.7% of patients operated on by laparoscopy). The occlusive events occurred at 1 month, 2 years, and 3 years. There was no difference in terms of recurrence or postoperative chronic pain. There were more seroma and mesh infections in the EP group (*p* < 0.05). Two patients operated on by laparoscopy had undetected bowel injuries, prompting emergent surgery for peritonitis. Conclusions: No statistically significant difference was found in terms of bowel obstruction between the intraperitoneal and the extraperitoneal position, but all cases of obstruction happened in the intraperitoneal mesh group. Visceral lesions remain a major complication of the laparoscopic approach that should not be neglected.

## 1. Introduction

Ventral hernias are one of the most frequent pathologies encountered. Despite the fact that they are highly prevalent, significant variability in their management exists. The variability is mainly due to both heterogenicity and a lack of high-quality evidence in the literature [1,2]. It has been evaluated that high-quality evidence such as randomized controlled trials about ventral hernia repair represents only 3% of the literature [1]. The majority include only case series and retrospective studies. This is why much disagreement exists between parietal experts in the management of ventral hernia repair. However, what is agreed on and solidified by high-quality evidence is the need for mesh reinforcement for ventral hernia repair [2]. A recent metanalysis of RCTs claimed that mesh reinforcement of umbilical hernia protected significantly against recurrence, with no increase in seroma formation, surgical site infection, hematoma, or chronic pain [3]. Even with this high-quality evidence, it has been estimated that mesh reinforcement is performed in less than 50% of ventral hernia repairs [2].

In current surgical practice, the mesh is essentially placed in an intraperitoneal (IP or IPOM: intraperitoneal onlay mesh) or extraperitoneal (EP) position. The EP position could either be in the preperitoneal or in the retromuscular plane. The term “sublay” is used for these two extraperitoneal planes. Although the use of a mesh at the time of surgery to prevent recurrences is already established, its position remains controversial [1,3,4,5,6]. The onlay position is agreed upon to be an unfavorable position as it has a higher rate of recurrence, seroma formation, and surgical site infection when compared to other positions [6]. Disagreements in the literature occur regarding the intraperitoneal position and extraperitoneal position as RCTs have proven a difference in terms of recurrence rates [6]. However, there is clear movement and agreement between experts that the optimal position is the EP [1,2,6].

Until the arrival of robot-assisted surgery, laparoscopic mesh placement was almost exclusively performed in an intraperitoneal position (laparoscopic IPOM). Robotic surgery allows the placement of a sublay mesh with a minimally invasive approach, therefore having the advantages of being both a minimally invasive approach and the sublay position. The main benefit of minimally invasive hernia repair is to reduce postoperative morbidity (namely surgical site infection) and to reduce the length of stay by improving postoperative recovery [7,8,9].

Different complications have been described in ventral hernia repair. Their frequency depends on the surgical approach, with laparoscopic surgery having a higher risk of visceral and vascular injuries [10], while open surgery has a higher risk of surgical site infection [11]. Other complications are related to the technique chosen to fix the mesh [12,13]. Finally, some are specifically related to the intraperitoneal mesh placement: mesh migration, intestinal incarceration between the mesh and abdominal wall, and intrabdominal adhesions leading to intestinal obstruction [14,15,16]. Furthermore, an American meta-analysis found higher intra-hospital costs with the laparoscopic technique [8].

Studies comparing the different techniques have mainly focused on their short-term results (safety, postoperative pain) and the recurrence rates [8]. However, the absence of a long-term follow-up looking for any further abdominal complication is a major drawback. To our knowledge, no study has evaluated the risk of long-term bowel obstruction in patients having an intraperitoneal mesh with their potential in creating adhesions. Herein, the aim of this study was to compare the likelihood of bowel obstruction according to the position of the mesh (either intraperitoneal or extraperitoneal) in a cohort of patients having undergone umbilical or periumbilical hernia repair.

## 2. Patients and Methods

This retrospective study included all patients operated on in the Digestive Surgery Department at the Dijon University Hospital, for primary ventral hernias, between March 2008 and July 2021. Exclusion criteria were epigastric, groin, or Spiegel hernias; recurrences; incisional hernias; hernia repair combined with another abdominal surgery; and primary repair with suture without leaving a mesh. All laparoscopic hernia repairs during this period were performed with an IPOM technique, placing an intraperitoneal composite mesh fixed with absorbable staples (Absorbatack, Medtronic, Dublin, Ireland).

The choice of mesh position and the surgical approach was left to the surgeon. However, since approximately 2018, the laparoscopic approach has been progressively abandoned in our center due to key opinion leaders in abdominal wall surgery. The preferred approach, therefore, is an open approach with a sublay mesh position. The laparoscopic approach was only proposed for severely obese patients, to avoid the higher risk of surgical site infections.

**Figure 1:**
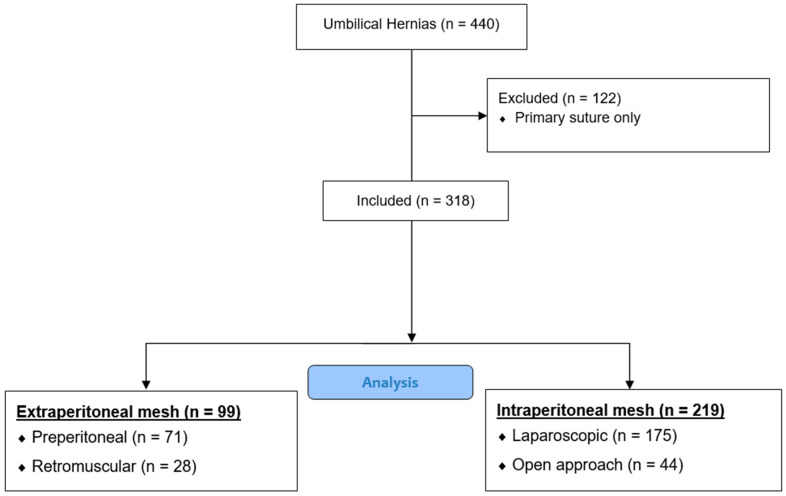
Flow chart of patients included.

Patients were divided into two groups according to the mesh position used: IP and EP. The IP mesh group had their mesh placed either by laparoscopy or with an open approach. During the inclusion period, no laparoscopic technique existed to place the mesh in the EP position; therefore, the EP mesh group was always performed in an open approach. Each patient received a single mesh. Figure 1 shows the flow chart.

Variables collected included demographic data (age, sex), past surgical history, weight, height, body mass index (BMI), previous symptoms, size of the hernia defect, and technique of repair (type of mesh, size, position, and fixation technique).

### 2.1. Endpoints and Follow-Up

The primary outcome was the occurrence of an episode of bowel obstruction requiring hospitalization and confirmed by abdominal CT scan. The management strategy, whether medical or surgical, was recorded.

Secondary endpoints were chronic postoperative pain beyond 1 month (defined as persistent pain after 1 month that required regular analgesics), bulging, recurrence, mesh infection, and the need for reoperation for any cause. Early postoperative complications (seroma, hematoma, wall infection, ileus) were also recorded.

Follow-up data were collected from the medical records of the postoperative regular visit at 6 weeks, and by a phone call later on to complete missing data and assess long-term results. Phone calls followed a standardized questionnaire (Figure S1 as a supplementary document). When needed, patients were asked to revisit the surgical department to be re-evaluated.

#### Ethical Approval

This was a retrospective study performed mainly on medical records. Therefore, ethical approval by a committee was not required. A phone call with patients was performed exclusively to update the follow-up and answer several questions, with a scientific goal. Therefore, specific approval for a phone call was not requested. However, all patients were informed that the phone call was for research purposes and oral approval was obtained before starting the questionnaire.

### 2.2. Statistical Analysis

Qualitative variables were presented as percentages and quantitative variables as mean and standard deviation (σ) or median and interquartile range (IQR). Qualitative data were compared with the Chi^2^ test and quantitative data with the Student *t* test, as data followed a normal distribution according to the histogram. The threshold of statistical significance was set with *p* < 0.05. Data were analyzed with the Excel (Microsoft Office version 16.74, Microsoft, Redmond, WA, USA) and R software (version 4.3.1).

## 3. Results

Between March 2008 and July 2021, 440 patients with primary umbilical or periumbilical hernias were operated on at the University Hospital of Dijon: 264 with an open approach and 176 by laparoscopy. Patients having undergone a simple suture of their hernia defect without using a mesh (122 patients) were excluded, leaving a total of 318 patients included in this study.

The EP group included 99 patients, in whom 71 meshes were placed in the preperitoneal plane and 28 meshes were placed in the retromuscular plane. The IP group included 219 patients, with 175 patients operated on laparoscopically, versus 44 patients by the open approach.

The median age of the patients was 51 years (IQR: 39–62), with a mean BMI of 28.9 kg/m^2^ (σ = 6). The follow-up duration was 7 years (IQR: 5–9) in the IP group and 3 years (IQR: 1–4.5) in the EP group.

### 3.1. Patient Characteristics

Demographic characteristics, hernia characteristics with their corresponding surgeries, surgical histories, and early postoperative characteristics are detailed in Table 1. Similar baseline characteristics were found between the two groups, except for the smoking history, which was significantly greater in the IP group (31%, *p* = 0.044). Both groups included more male patients than females. Similarly, in the IP group, there were similar baseline characteristics in both patients operated on by the open approach and laparoscopically. The rate of emergency surgery was significantly higher in the EP group (13.13%) than IP (13.13% vs. 4.5%, *p* < 0.05). Symptoms leading to consultation were, in order of frequency, discomfort, pain, incarceration, and skin disorders.

The median size of hernial defects was 20 mm (IQR 10–26.3). Patients operated on with an IP technique had a significantly smaller hernial defect (mean 19 mm, σ: 11.5) than patients with an EP mesh (25 mm, σ: 16.8) (*p* < 0.05). During the laparoscopic approach, 21.7% of hernial defects were closed before mesh placement.

Parietex™ (Medtronic, Paris, France) constituted almost half (47.5%) of the all meshes placed, followed by 21% CA.B.S. Air^®^ (Cousin, Wervicq-Sud, France) and Prolene^®^ (Ethicon, Cincinnati, OH, USA) in 8% of the patients. Others, in order of frequency, were SURGIMESH^®^, Phasix (BD, Franklin Lakes, NJ, USA), ProGrip^®^ (Medtronic, Minneapolis, MN, USA), Vicryl^®^ (Ethicon, Laritan, NJ, USA), Dualmesh^®^ (Gore, Newark, DE, USA), and Physiomesh^®^ (Johnson & Johnson, New Brunswick, NJ, USA). Table 2 summarizes the types of meshes used, according to their positions.

### 3.2. Primary Endpoint

Among all patients included in the study, three patients presented an episode of acute intestinal obstruction during the follow-up period (at 1 month, 2 years, and 3 years). All three patients had initially undergone a laparoscopic repair with an IP mesh (Table 3). Two had a Parietex^®^ mesh, and one had a Surgimesh^®^ mesh. No patient with an intraperitoneal mesh operated on by an open technique, or those with an EP mesh, presented an occlusive crisis. The difference between the IP and the EP group was not significant (*p* = 0.245). Among these three patients, only one, with a Parietex^®^ mesh, required surgical management of the occlusion (no bowel resection required) due to significant adhesions on the mesh, while the two other patients were treated conservatively. Each of these patients had a single episode of bowel obstruction.

### 3.3. Secondary Endpoints

Thirty-nine patients in the IP group (17.8%) had postoperative pain beyond one month after surgery, versus nine patients in the EP group (9%), the difference being at the limit of significance (*p* = 0.079). There was no difference in terms of recurrence (10.9% in the IP group versus 12.1% in the EP group, *p* = 0.787) or the impression of bulging in either group (*p* = 0.202). Concerning mesh infections, four patients (1.8%) in the IP group presented an infection, against eight patients (8%) in the EP group, this difference being significant (*p* = 0.009), with a consequently higher rate of revision surgery in the EP group (12.1% of patients against 4.1% of patients in the IP group) (*p* < 0.05).

No postoperative ileus was found in either group; there was no difference between groups in terms of hematoma (4.1% in the IP group vs. 6.1% in the EP group) or superficial infection (5.4% in the IP group vs. 12.1% in the EP group). The frequency of seroma formation was significantly higher in the EP group (6%) as compared to the IP group (0.9%; *p* < 0.05).

Interestingly, two patients in the IP group (both operated on by laparoscopy) had an undetected bowel injury prompting emergent surgery for peritonitis. The first patient required surgical revision with a simple suture of two perforations; the postoperative phase was uneventful. The second patient required a two-stage surgery because of septic shock with resection and anastomosis of the small intestine and a 48-h stay in the intensive care unit. The frequency of this complication, however, did not reach statistical significance (*p* = 0.342).

## 4. Discussion

We performed a retrospective study of all patients operated in the University Hospital of Dijon, on their primary ventral hernias, comparing the IP and EP mesh placements. The main aim of the study was to asses the frequencies of bowel obstruction for those having an IP and EP mesh placement, regardless of the surgical approach. The study found three patients with an IP mesh who presented with an occlusive crisis during follow-up. The difference did not reach statistical significance due to the low number of patients. Furthermore, two patients in the IP group operated on by the laparoscopic approach were found to have an undetected bowel injury that required urgent surgical repair.

No study in the literature has examined the long-term risk of bowel obstruction in patients with intraperitoneal meshes, despite several authors claiming that they cause adhesions as compared to extraperitoneal meshes. The widespread use of the laparoscopic repair of ventral hernias explains the ongoing placement of intraperitoneal meshes. The simplicity of the procedure, and especially its cosmetic outcome, is favored by many surgeons. This is especially the case in private practice, where cosmetic issues, operative times, and postoperative outcomes play a major role in the selection of a surgical strategy [17]. The IP placement of a mesh laparoscopically or by the open approach is much faster and less demanding than the EP plane, but carries a risk of adhesion, migration, pain, and occlusion [12,18,19,20]. Even with experts’ opinion favoring the extraperitoneal plane of mesh placement, the intraperitoneal mesh placement is still being performed.

A recent study on experimental animal models showed that all intraperitoneal meshes, despite a special anti-adhesion coating, were associated with the development of adhesions and thus with a potential risk of intestinal obstruction or long-term postoperative pain [21,22]. This is also what is usually noted by surgeons reoperating on patients having an intraperitoneal mesh already placed [17]. Whether in an emergency setting or for other pathologies, when reoperating on a patient with an intraperitoneal mesh, the finding of intraperitoneal adhesions with the mesh is almost guaranteed [17,19]. In our experience, these procedures can be challenging as the operative time is significantly prolonged and the risk of bowel injury is potentially increased.

Supporting the actual agreement of experts in shifting from IP to EP mesh placement is therefore the potential risk of bowel obstruction, as well as other well-known complications of IP mesh placement, such as fistula, mesh migration, chronic pain, and difficulties at the time of reoperation [12,17,18,19]. It is estimated that in the case of laparoscopic reoperation in patients with an IP mesh, the relative risk of adverse events is four times that of a patient without an IP mesh [17]. Despite the improvement in the quality of composite meshes, with the claim of being non-adherent, the ideal intraperitoneal mesh is still to be found [19]. This is due to the fact that, in our experience, most if not all intraperitoneal composite meshes create intraperitoneal adhesions postoperatively, complicating further surgical intervention when necessary. This fact has been claimed similarly by other studies, where adhesions after intraperitoneal mesh placement have been noted [19,21,22,23].

All three cases of bowel obstruction in this study occurred in those having an IP mesh placement. All were operated on by laparoscopy, with two patients having a Parietex and one Surgimesh mesh placement. Due to the low number of patients in this study, no significance was reached, neither for the mesh position nor for the mesh type. Other studies have claimed that regardless of the mesh used, whether synthetic or biological, all types induce intraperitoneal adhesions [19,23]. Furthermore, laparoscopic procedures are known to induce less intraperitoneal adhesions than the open approach; therefore, the adhesions in patients with IP meshes could only be attributed to the mesh itself [19,20].

The difference in terms of pain beyond one month showed also a trend related to the IP mesh, but the difference did not reach significance. Postoperative pain after hernia repair is another subject that has been widely studied in the literature [12,19,20]. Especially after a laparoscopic hernia repair with IP mesh placement, the type of mesh fixation plays a major role in postoperative pain [12]. Adequate fixation is mandatory to stabilize the mesh, especially during the integration phase postoperatively, to ensure the best results. It must, however, prevent the contact of the adhesive non-protective part of the mesh with the intraperitoneal viscera. The laparoscopic technique was initially described using mainly staples or tackers to fix the mesh in place. Due to high recurrence rates, and the risk of bowel incarceration between a loose part of the mesh and the abdominal wall, some surgeons started to add transabdominal stay sutures, in addition to the staples, to stabilize further the IP mesh [12]. Gradually, a concern for postoperative discomfort and pain arose, due to the possible risk of the transabdominal sutures causing nerve and vessel entrapment [7,12,18]. The risk of postoperative pain and discomfort plays presently an important role in deciding which surgical technique to adopt for ventral hernia repair. Lastly, since parietal surgeons are usually confident in their own techniques, changing the way in which the mesh is fixed represents a dilemma and a source of disagreement between experts. This highlights the need for high-quality evidence and strict recommendations.

The benefit of minimally invasive procedures in reducing the risk of mesh infection and the seroma rate is well known in the literature [8,24,25,26]. Our study confirms this advantage, with a significantly higher frequency of surgical site infections, with no difference found concerning hematomas. However, serious complications specific to laparoscopy, notably visceral injury with subsequent peritonitis, are present. Although their frequency did not reach statistical significance due to the small population size in our study, their clinical impact is major and there is no doubt that these complications are specific to the laparoscopic approach [8,24].

Ideally, the combination of the benefits of a minimally invasive procedure (in particular, recovery and the lower risk of infection) and EP mesh placement seems to offer a solution to prevent the risk of adhesion while preserving the benefit of the minimally invasive approach [25,26]. This would support the choice of certain teams to carry out laparoscopic ventral hernia repair with a sublay mesh [27,28]. However, this technique is difficult and prolongs the operative time. The promising contribution of robotic surgery could resolve this issue [29,30]. However, regardless of the technique, minimally invasive procedures will always remain associated with a higher risk of rare but life-threatening injuries, such as visceral or vascular ones [10].

There were several limitations to this study. The retrospective nature of this study implies that there were likely some missing data during data collection. Some data were not easily found in patients’ files. This was resolved by asking further questions during the phone calls with patients to complete such data. Some difficulties were faced during the phone calls with patients. Some patients had difficulties in expressing their symptoms, which required us at times to ask the patient to revisit the outpatient clinic. A minority of patients had a language barrier, which was resolved with the help of a translator.

## 5. Conclusions

This study compared the likelihood of acute bowel obstruction after primary umbilical or periumbilical hernia repair according to the position of the mesh. No statistically significant difference was found in terms of bowel obstruction between the intraperitoneal and the extraperitoneal position, but all cases of obstruction happened in the intraperitoneal mesh group. Bowel injury during laparoscopic hernia repair remains an unneglectable and severe complication related to minimally invasive procedures. Bowel obstruction, in addition to all well-known complications of IP mesh placement, supports experts’ opinions about abandoning IP mesh placement and supporting the EP plane. Furthermore, a large prospective study should assess the risk of bowel obstruction after IP mesh placement.

## Figures and Tables

**Table 1:** Characteristics, size of hernia, post operative complications, and follow up of patients included in the study.

N = 318		IP 219		EP 99		*p*
		n	%	n	%	
Gender	Male	123	56	60	61	
	Female	96	44	39	39	0.635
Age (mean)		49.67	-	53.04	-	0.066
Weight (mean)		84.41	-	86.31	-	0.445
BMI (mean)		28.89	-	29.11	-	0.783
Obesity		73	33.3	43	43.43	0.243
Smoking		68	31.0	17	17.17	0.044
Surgical History		35	15.9	15	15.15	0.872
Emergency Surgery		10	4.5	13	13.13	0.012
Size of the Defect (mean in mm)		19.07	-	25.45	-	0.001
Early Complications						
Seroma		2	0.9	6	6.06	0.008
Hematoma		9	4.1	6	6.06	0.470
Wall Infection		10	5.4	12	12.12	0.163
Ileus		0	0	0	0	
Bulging		27	12.3	7	7	0.202
Intestinal Obstruction		3	1.4	0	0	0.245
Small Bowel Perforation		2	0.9	0	0	0.342
Follow-up (years)		7 IQR (5–9)		3 IQR (2–4.5)	-	-

**Table 2:** Types of meshes according to their position.

	Intraperitoneal (n = 219)		Extraperitoneal (n = 99)		Total	
	N	%	N	%	N	%
Parietex™	143	65.3	8	8.0	151	47.48
CA.B.S.’ Air^®^	20	9.13	47	47.47	67	21
Prolene	8	3.65	19	19.19	27	8.49
SURGIMESH WN	23	10.5	0	0	23	7.23
ProGrip™	0	0	14	14.14	14	4.71
PHASIX^®^ or Phasix ST	1	0.45	9	9.09	10	3.14
Vicryl	6	2.74	0	0	6	1.88
Others (DUALMESH^®^, Physiomesh^®^)	4	1.83	1	1.01	5	1.5
Not specified	14	6.39	1	1.01	15	4.71

**Table 3:** Characteristic and management of patient that presented with intestinal obstruction.

Age	Mesh Type	Mesh Position	Surgical Approach	Follow up until IO	Management
64	Surgimesh	Intraperitoneal	Laparoscopic	1 month	Conservative
50	Parietex	Intraperitoneal	Laparoscopic	2 years	Conservative
31	Parietex	Intraperitoneal	Laparoscopic	3 years	Surgery

IO = Intestinal Obstruction.

## Data Availability

Data could be provided upon request by the corresponding author.

## Supplementary Materials

The following supporting information can be downloaded at: https://www.mdpi.com/article/10.3390/jcm12165341/s1, Figure S1: Standardized Questionnaire.
