# Supplementary material for: Primary Ventral Hernia Repair and the Risk of Postoperative Small Bowel Obstruction: Intra Versus Extraperitoneal Mesh
Source: J Clin Med. 2023 Aug 16;12(16):5341. doi: 10.3390/jcm12165341 (PMC10455485; doi:10.3390/jcm12165341)
Supplement: Supplementary file 1 [file jcm-12-05341-s001.zip › jcm-2477084-supplementary.pdf]

QUESTION	YES	NO
Do you have chronic abdominal pain?		
Do you feel a bulging at the site of hernia repair?		
Did any medical professional confirm a recurrence of your hernia?		
Did you have a mesh infection proven by a medical professional?		
Did you undergo a re-do surgery for recurrence?		
Did you have any symptoms of intestinal obstruction (detailed)		
If yes was it treated conservatively		
If yes was it treated surgically		

Figure S1. Standardized Questionnaire.
